# Damaged Self-Esteem is Associated with Internalizing Problems

**DOI:** 10.3389/fpsyg.2013.00152

**Published:** 2013-04-02

**Authors:** Daan H. M. Creemers, Ron H. J. Scholte, Rutger C. M. E. Engels, Mitchell J. Prinstein, Reinout W. Wiers

**Affiliations:** ^1^Mental Health Care InstituteGGZ Oost-Brabant, Veghel, Netherlands; ^2^Behavioural Science Institute, Radboud University NijmegenNijmegen, Netherlands; ^3^University of North Carolina at Chapel HillChapel Hill, NC, USA; ^4^Addiction, Development, and Psychopathology-Lab, Department of Psychology, University of AmsterdamAmsterdam, Netherlands

**Keywords:** damaged, implicit cognition, self-esteem, suicidal ideation, depression, loneliness

## Abstract

Implicit and explicit self-esteem are assumed to be important factors in understanding the onset and maintenance of psychological problems. The current study aims to examine the association between implicit and explicit self-esteem and their interaction with depressive symptoms, suicidal ideation, and loneliness. Specifically, the relationship between the *size* and the *direction* of the discrepancy between implicit and explicit self-esteem with depressive symptoms, suicidal ideation, and loneliness were examined. Participants were 95 young female adults (*M* = 21.2 years, SD = 1.88) enrolled in higher education. We administered the IAT to assess implicit self-esteem, and the Rosenberg self-esteem scale to measure explicit self-esteem while psychological problems were assessed through self-reports. Results showed that discrepancies between implicit and explicit self-esteem were positively associated with depressive symptoms, suicidal ideation, and loneliness. In addition, the direction of the discrepancy was specifically relevant: damaged self-esteem (i.e., high implicit self-esteem and low explicit self-esteem) was consistently associated with increased levels of depressive symptoms, suicidal ideation, and loneliness. In contrast, defensive or fragile self-esteem (i.e., low implicit and high explicit self-esteem) was solely associated with loneliness. These findings provide further support that specifically damaged self-esteem is an important vulnerability marker for depressive symptoms, suicidal ideation, and loneliness.

## Introduction

Self-esteem plays a crucial role in the onset and maintenance of internalizing problems (Harter, 1993; Brage and Meredith, 1994; Prinstein and La Greca, 2002; Evans et al., 2004). Research has mainly focused on the association of explicit self-esteem with internalizing problems, whereas there is growing evidence that implicit self-esteem might be an important construct to examine in relation to internalizing problems. Implicit self-esteem is defined as relatively automatic, overlearned, and non-conscious evaluation of the self that guides spontaneous reactions to self-relevant stimuli (Greenwald and Banaji, 1995). Implicit self-esteem is a complex, multi-dimensional construct (Koole and Pelham, 2003) and, therefore, it has been argued that various measures of implicit self-esteem may be addressing different facets of this construct. Moreover, in the field of research on implicit and explicit self-esteem results often show that, in addition to its unique associations, the discrepancy between implicit and explicit self-esteem is considered to be relevant for understanding psychopathology. A recent study (Creemers et al., 2012) showed that discrepancies between implicit and explicit self-esteem were positively associated with depressive symptoms, suicidal ideation, and loneliness. In addition, the direction of the discrepancy has been found to be specifically relevant: damaged self-esteem (high implicit self-esteem and low explicit self-esteem) was related to increased levels of depressive symptoms, suicidal ideation, and loneliness, while defensive or fragile self-esteem (low implicit self-esteem and high explicit self-esteem) was not.

In order to understand how asymmetric changes between implicit and explicit self-esteem develop, dual process models provide an useful theoretical framework. According to recent dual process models, two distinct information-processing modes with different operating principles can be distinguished: the reflective and the associative mode (Epstein, 1994; Gawronski and Bodenhausen, 2006). Explicit self-esteem reflects a product of the reflective mode, shaped through rational and conscious processing of self-relevant stimuli, whereas implicit self-esteem is assumed to be the outcome of the associative mode, shaped through more automatic, intuitive, unconscious processing of affective experiences (Epstein and Morling, 1995; Dijksterhuis, 2006). As a result of the distinct cognitive processes asymmetric changes (for example, decrease in explicit self-esteem but not in implicit self-esteem) between implicit and explicit self-esteem may occur. It has been proposed that damaged self-esteem in depressed individuals may represent a discrepancy between “the ideal self” and “the actual self.” Implicit self-esteem, which is proposed to develop earlier in interaction with primary care givers (e.g., DeHart et al., 2006), may be indicative for the “ideal self.” Subsequently, explicit self-esteem may be indicative for the more recently formed “actual self.” As a result of the discrepancy people may feel entrapped between their goals and “reality” which in turn may lead to internalizing problems.

Two common measures to assess implicit self-esteem are the Name Letter Task (NLT) and the Implicit Association Test (IAT). Implicit self-esteem as conceptualized by the NLT is supposed to assess an aspect of implicit self-esteem that differs from the IAT and, therefore, these two measures are not correlated with each other (Bosson et al., 2000). Importantly, implicit self-esteem is defined as the relatively automatic, overlearned, and non-conscious evaluation of the self that guides spontaneous reactions to self-relevant stimuli (Greenwald and Banaji, 1995). The finding that both measures of implicit self-esteem are not related might be due to the distinct self-relevant stimuli that are being used in the IAT and NLT. To illustrate, the IAT measures the strength of the association between “the self” and “worthless” or “valuable,” while the NLT asses the relative preference for one’s own initials assuming these stem from self-associations in memory. Although previous studies showed that both measures of implicit self-esteem were not correlated (Bosson et al., 2000), similar associations between the two measures and internalizing problems were found (e.g., Franck et al., 2007; Creemers et al., 2012). This might indicate that both aspects of implicit self-esteem are part of an analogous underlying mechanism that is associated with the onset and development of internalizing problems. Therefore, in order to gain a full perspective of the associations between implicit self-esteem and internalizing problems the use of multiple implicit measures is relevant.

In the Creemers et al. (2012) paper implicit self-esteem was measured with the NLT. In the present study we will report on the associations of implicit self-esteem and explicit self-esteem (and their discrepancy) with psychopathology using different implicit measures. The measures we used to assess implicit self-esteem were two versions of the IAT (Greenwald et al., 1998; Sriram and Greenwald, 2009). Our aim was to test whether the findings reported in Creemers et al. (2012) could be extended by using these different measures of implicit self-esteem. We hypothesized that specifically damaged self-esteem was associated with internalizing problems.

## Materials and Methods

### Participants

All participants volunteered to participate in the study after informed consent. Participants were 95 female undergraduate students of a College for Higher Professional Education, in The Netherlands^1^. Their mean age was 21.2 years (SD = 1.88, Range = 19–30).

### Procedure

Prior to the data collection all participants were told that the experiment examined various predictors of human emotion. First, a computerized implicit measure, the IAT was administered. After completing this task, the computerized explicit self-esteem scale was assessed. Next, participants completed questionnaires on depressive symptoms, suicidal ideation, and loneliness.

### Measures

#### Explicit self-esteem

The Rosenberg self-esteem scale (RSES; Rosenberg, 1965) was used to assess global feelings of self-esteem (e.g., “I feel I do not have much to be proud of”). This self-report questionnaire consists of 10 items measured on a 4-point scale (*totally agree* – *totally disagree*). Validity and test-retest reliability of the RSES are satisfactory (Franck et al., 2008). Cronbach’s α was 0.87 for the present sample.

#### Implicit self-esteem

##### Implicit association test

The IAT measures associations between four categories by pairing two target categories (i.e., me/not-me) with two attribution categories (i.e., valuable/worthless; Greenwald et al., 1998). The underlying assumption of the IAT is that when certain concepts (i.e., valuable and me) are more strongly associated in memory than other concepts (i.e., valuable and not-me), responses are faster when these concepts share a response key. Thus, the faster the response time, the stronger the presumed association is between two categories in memory. In our study, “valuable” and “me” sharing one response key, and “worthless” and “not-me,” sharing another response key, was the compatible block. The incompatible block consisted of “valuable” and “not-me” sharing one response key, and “worthless” and “me” sharing another response key. The mean difference in reaction times between compatible and incompatible trials is used to estimate the IAT effect: the relative associative strength between the two pairs of concepts. The IAT consisted of seven blocks of trials, and similar stimuli were used as described by Franck et al. (2007). The improved scoring algorithm (Greenwald et al., 2003) was used (D600 algorithm) to compute the individual effect size of the subjects. In our study, higher scores indicated higher levels of implicit self-esteem. Past research demonstrated satisfactory reliability and validity of the IAT (Greenwald et al., 1998; Nosek et al., 2005). The IAT was programed in Inquisit 3.0 (Millisecond software). As an index of internal consistency for the IAT we computed the correlation between the test and practice blocks. A significant correlation was found (*r* = 0.62; *p* < 0.001).

#### Depressive symptoms

A Dutch version of the *Beck Depression Inventory* (*BDI-II*; Beck et al., 1996; Van der Does, 2002) was administered to measure the severity of depressive symptoms. Satisfactory validity and psychometric properties of this scale were demonstrated in previous research (Van der Does, 2002). This self-report questionnaire consists of 21 items assessed on a 4-point scale, with items ranging from 0 to 3. Cronbach’s α was 0.89.

#### Suicidal ideation

Participants completed a Dutch version of the questionnaire developed by Heilbron and Prinstein (2010) to measure suicidal ideation. This self-report measure assesses suicidal thoughts in adolescents and young adults (e.g., “I thought that killing myself would solve my problems”). This scale consists of 16 items measured on a 5-point scale (*never* – *almost every day*), and includes a subset of items drawn from the Suicidal Ideation Questionnaire (SIG; Reynolds, 1988) and the NIMH-DISC-IV (Shaffer et al., 2000). Cronbach’s α was 0.89.

#### Loneliness

A short version (R-ULS-8, Roberts et al., 1993) of the revised UCLA Loneliness Scale (R-ULS; Russell et al., 1980) was used to assess loneliness. Satisfactory construct validity and reliability were found in other adolescent populations (Higbee and Roberts, 1994). This instrument consists of 8-items (e.g., “I feel left out”) assessed on a 5-point scale (*I totally disagree* – *totally agree*). Cronbach’s α was 0.84.

### Data analyses

First, a series of hierarchical multiple regression analyses were performed to examine the relationship between implicit, explicit self-esteem, and their interaction with suicidal ideation, depressive symptoms, and loneliness. Implicit and explicit self-esteem were entered in step 1 and their interaction in step 2. Second, we examined the relationship of implicit-explicit discrepancies with depressive symptoms, suicidal ideation, and loneliness. The absolute difference between the standardized score on implicit and explicit self-esteem was computed, which indicated the *size* of the discrepancy. A higher score on this variable was indicative for a larger implicit-explicit self-esteem discrepancy. Next, a dummy variable was computed to determine the direction of the discrepancy between implicit and explicit self-esteem (implicit < explicit or implicit > explicit; dummy code). In the present study, 49 participants showed higher implicit than explicit self-esteem, and 46 participants reported higher explicit than implicit self-esteem. In order to examine whether implicit-explicit self-esteem discrepancies were related to suicidal ideation, depressive symptoms, and loneliness, a series of hierarchical multiple regression analyses were performed. The *size* of the discrepancy and the *direction* of the discrepancy (dummy) were entered in step 1 and their interaction in step 2. As argued in several recent papers (Briñol et al., 2006; Schröder-Abé et al., 2007; Creemers et al., 2012), these discrepancy analyses are an appropriate manner of specifically testing the associations of implicit-explicit self-esteem discrepancies with internalizing problems.^2^ Interactions were tested using the procedure proposed by Aiken and West (1991).

## Results

### Intercorrelations among the measures

Descriptive statistics of all study variables were presented in Table 1. The intercorrelations among all study measures are displayed in Table 2. The measures of implicit and explicit self-esteem were weakly correlated. Next, explicit self-esteem was negatively correlated to depressive symptoms, suicidal ideation, and loneliness. Depressive symptoms, suicidal ideation, and loneliness were positively correlated.

**Table 1:** **Descriptive statistics for measures of implicit and explicit self-esteem, depressive symptoms, suicidal ideation, and loneliness**.

	Mean	SD	Range	Min	Max
Impl. self-esteem (IAT)	0.79	0.47	***	−0.81	1.86
Expl. self-esteem	30.65	4.42	10–40	18.00	40.00
Depressive symptoms	8.29	7.32	00–63	0.00	34.00
Suicidal ideation	8.47	1.72	16–80	8.00	21.00
Loneliness	14.49	5.46	8–40	8.00	30.00

**Table 2:** **Correlations among measures of implicit and explicit self-esteem, depressive symptoms, suicidal ideation, and loneliness**.

	1	2	3	4	5
Impl. self-esteem (IAT)	–				
Expl. self-esteem	0.23*	–			
Depressive symptoms	−0.23*	−0.70**	–		
Suicidal ideation	0.05	−0.36**	0.38**	–	
Loneliness	−0.24*	−0.67**	0.60**	0.29**	–

*p < 0.05; **p < 0.01.

In addition to the conventional IAT, the Brief IAT (Sriram and Greenwald, 2009) was administered to assess implicit self-esteem. A significant correlation was found between the IAT and Brief IAT (*r* = 0.48^**^). Results of the analyses with the Brief IAT are similar as the presented results with the IAT, and therefore only available in an online Appendix.

### Associations with explicit and implicit self-esteem

As presented in Table 3, results of step 1 show that explicit self-esteem significantly predicts unique variance in depressive symptoms (β = −0.69, *p* < 0.001), suicidal ideation (β = −0.44, *p* < 0.001), and loneliness (β = −0.65, *p* < 0.001). No significant associations of implicit self-esteem were found with depressive symptoms (β = −0.06, *p* = 0.40) suicidal ideation (β = 0.17, *p* = 0.08), and loneliness (β = −0.08, *p* = 0.31).

**Table 3:** **Hierarchical multiple regression analyses: associations of explicit self-esteem, implicit self-esteem, and the Interaction between implicit and explicit self-esteem with suicidal ideation, depressive symptoms, and loneliness**.

	Suicidal ideation			Depressive symptoms			Loneliness		
	B	SE	β	B	SE	β	B	SE	β
**STEP 1**									
Implicit self-esteem	0.01	0.01	0.17	−0.06	0.08	−0.06	−0.08	0.08	−0.08
Explicit self-esteem	−0.03	0.01	−0.44**	−0.69	0.08	−0.69**	−0.65	0.08	−0.65**
**STEP 2**									
Implicit self-esteem*	−0.01	0.01	−0.10	0.05	0.06	0.07	0.04	0.06	0.06
Explicit self-esteem									

Suicidal ideation *R*^2^ = 0.18 in step 1 (*p* = 0.00); Δ*R*^2^ = 0.01 in step 2 (*p* = 0.31); depressive symptoms *R*^2^ = 0.49 in step 1 (*p* = 0.00); Δ*R*^2^ = 0.01 in step 2 (*p* = 0.35); loneliness *R*^2^ = 0.46 in step 1 (*p* = 0.00); Δ*R*^2^ = 0.00 in step 2 (*p* = 0.49); *p < 0.05 **p < 0.01.

### Associations of the interaction between implicit and explicit self-esteem

In step 2 we entered the interaction between implicit and explicit self-esteem. Results showed no significant associations of the interaction between implicit and explicit self-esteem with depressive symptoms (β = 0.07, *p* = 0.35), suicidal ideation (β = −0.10, *p* = 0.30), and loneliness (β = 0.06, *p* = 0.50). Table 3 summarizes the results of the multiple hierarchical regression analyses.

### Associations of implicit-explicit discrepancies

**Figure 1:**
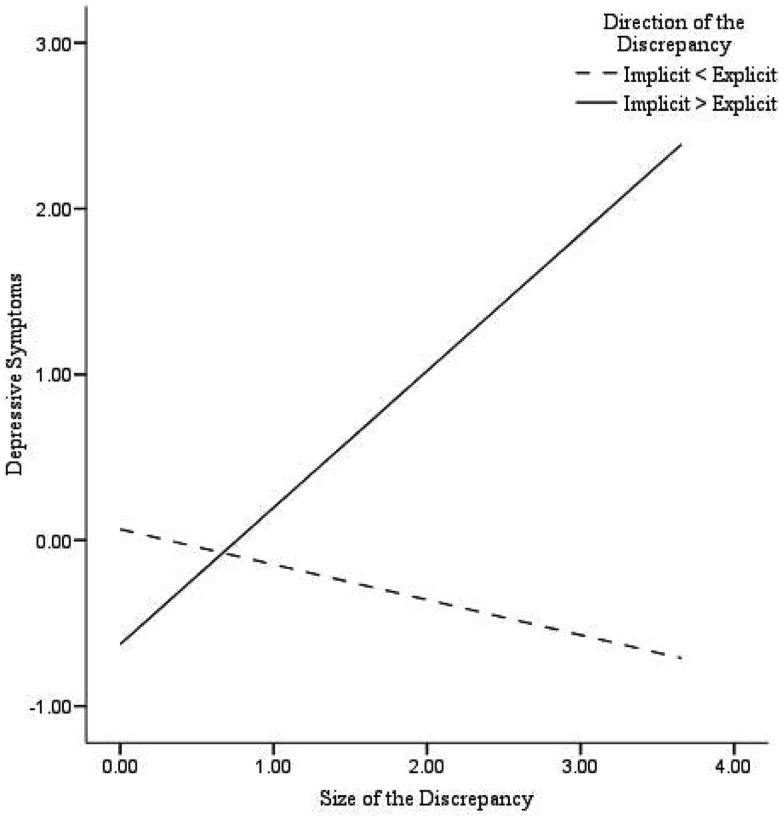
**Predicted values for depressive symptoms, illustrating the interaction between the size of the discrepancy and the direction of the discrepancy**.

**Figure 2:**
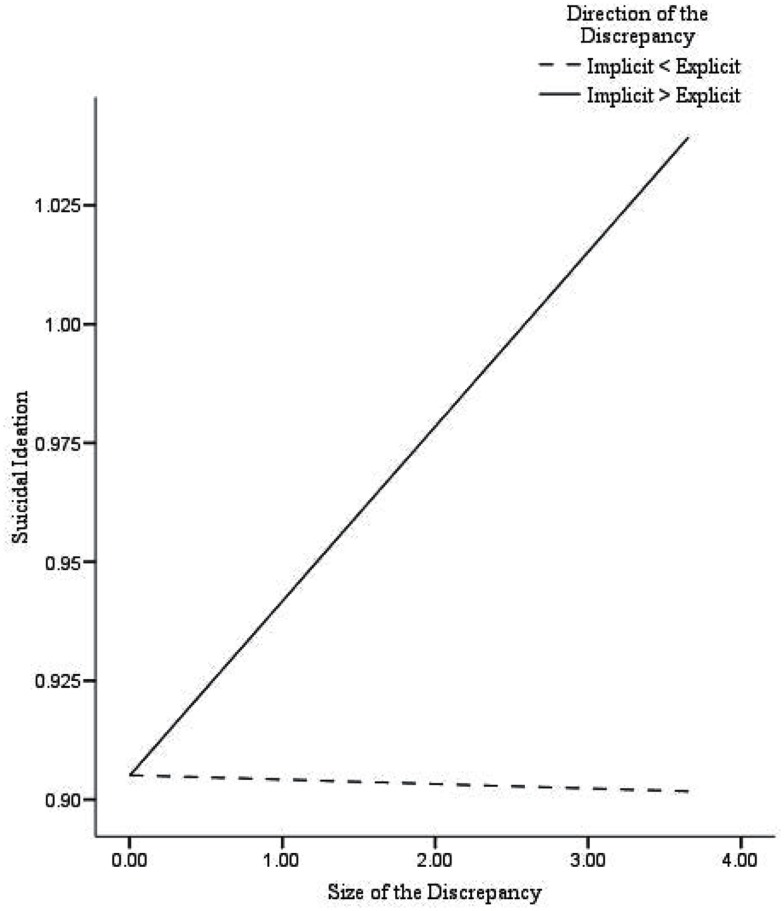
**Predicted values for suicidal ideation, illustrating the interaction between the size of the discrepancy and the direction of the discrepancy**.

**Figure 3:**
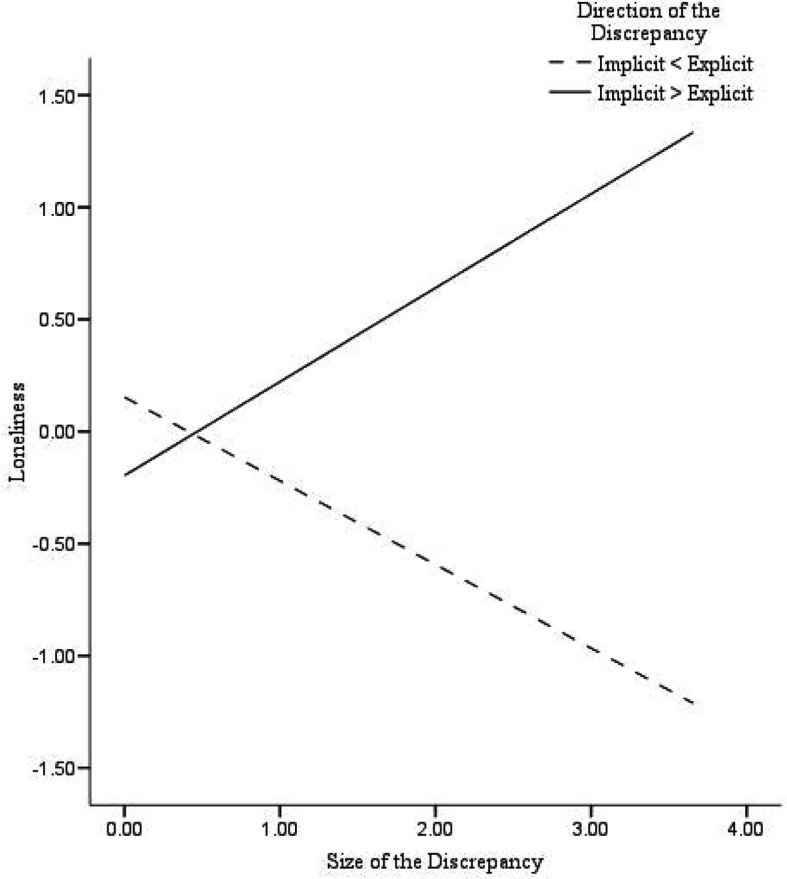
**Predicted values for loneliness, illustrating the interaction between the size of the discrepancy and the direction of the discrepancy**.

First, in participants with damaged self-esteem we found significant correlations between the *size* of the discrepancy and depressive symptoms (*r* = 0.68^**^), suicidal ideation (*r* = 0.31*), and loneliness (*r* = 0.35*), while in participants with fragile self-esteem no significant correlations were found. Second, a series of multiple hierarchical regression analyses were performed with the *size* of the discrepancy and the *direction* of the discrepancy (dummy coded) entered in step 1, and their interaction entered in step 2 (see text footnote 2). As shown in Table 4, the size of the discrepancy was positively associated with depressive symptoms (β = 0.28, *p* < 0.01), and suicidal ideation (β = 0.25, *p* = 0.01). There was no significant relationship of the size of the discrepancy with loneliness (β = 0.05, *p* = 0.66). The direction of the discrepancy was significantly associated with suicidal ideation (β = 0.28, *p* < 0.01), whereas no associations with depressive symptoms (β = 0.14, *p* = 0.16) or loneliness (β = 0.20, *p* < 0.06) were found. Moreover, the interaction between the *size* of the discrepancy and the *direction* of the discrepancy was related to depressive symptoms (β = 0.77, *p* < 0.001), suicidal ideation (β = 0.45, *p* < 0.05), and loneliness (β = 0.60, *p* < 0.01). Similarly, as in Creemers et al. (2012), significant associations were found between the *size* of the discrepancy and all measured internalizing problems in participants with damaged self-esteem (higher implicit then explicit self-esteem). Participants with fragile self-esteem (higher explicit then implicit self-esteem) showed no significant associations between the *size* of the discrepancy and depressive symptoms or suicidal ideation. However, we did find that the *size* of the discrepancy was negatively associated with loneliness in participants with fragile self-esteem. In sum, these findings indicate that damaged self-esteem is related to higher levels of depressive symptoms, suicidal ideation, and loneliness, whereas fragile self-esteem is solely related to lower levels of loneliness (Figures 1–3).

**Table 4:** **Hierarchical multiple regression analyses: associations of the size of the discrepancy, direction of the discrepancy, and the interaction between the size of the discrepancy and the direction of the discrepancy with suicidal ideation, depressive symptoms, and loneliness**.

	Suicidal ideation			Depressive symptoms			Loneliness		
	B	SE	β	B	SE	β	B	SE	β
**STEP 1**									
Size of the discrepancy	0.02	0.01	0.25*	0.35	0.13	0.28**	0.06	0.13	0.05
Direction of the discrepancy	0.04	0.01	0.28**	0.28	0.20	0.14	0.39	0.21	0.20
**STEP 2**									
Size of the discrepancy*	0.04	0.02	0.45*	1.04	0.23	0.77**	0.79	0.25	0.60**
Direction of the discrepancy									

Suicidal ideation *R*^2^ = 0.14 in step 1 (*p* = 0.00); Δ*R*^2^ = 0.06 in step 2 (*p* = 0.01); depressive symptoms *R*^2^ = 0.09 in step 1 (*p* = 0.01); Δ*R*^2^ = 0.17 in step 2 (*p* = 0.00); loneliness *R*^2^ = 0.04 in step 1 (*p* = 0.15); Δ*R*^2^ = 0.10 in step 2 (*p* = 0.00); *p < 0.05 **p < 0.01. Direction of the discrepancy was dummy coded: 0 = fragile self-esteem; 1 = damaged self-esteem.

## Discussion

The main purpose of the present study was to test whether recent findings regarding implicit self-esteem and the discrepancy between implicit and explicit self-esteem as concurrent predictors of internalizing problems (Creemers et al., 2012) could be extended by using a different measure of implicit self-esteem. Additional measures to assess implicit self-esteem (i.e., IAT and Brief IAT) were administered and used to validate our findings. Results showed that explicit self-esteem was negatively associated with depressive symptoms, suicidal ideation, and loneliness, whereas no unique associations of this assessment of implicit self-esteem with internalizing problems were found. Next, the relationship of the *size* and the *direction* of the discrepancy between implicit and explicit self-esteem, and their interaction with depressive symptoms, suicidal ideation, and loneliness were examined. As expected, results showed that the size of the discrepancy was positively associated with all indices of internalizing problems, specifically, in participants with damaged self-esteem (higher implicit than explicit self-esteem). In addition, for participants with defensive or fragile self-esteem (high explicit and low implicit self-esteem) we found that the size of the discrepancy was negatively associated with loneliness. Importantly, these findings indicate that damaged self-esteem is an important vulnerability marker for the onset and development of internalizing problems.

Overall, these findings confirm previous results that were presented in Creemers et al. (2012), however, they also extend current literature in several aspects. More specifically, our finding that damaged self-esteem is associated with depressive symptoms, suicidal ideation, and loneliness when implicit self-esteem is measured with the IAT is relevant. This further supports the assumption that discrepancies between implicit and explicit self-esteem are important to consider for understanding internalizing psychopathology. Subsequently, we think that current findings emphasize the use of implicit measures to examine (implicit) cognitive processes in relation to the maintenance and treatment of internalizing problems. For example, the therapeutic effect of Cognitive Behavior Therapy (CBT) might be different for individuals with discrepancies between implicit and explicit self-esteem. The enhancement of explicit self-esteem might be useful for individuals with damaged self-esteem (high implicit and low explicit), whereas it might be disadvantageous for individuals with fragile self-esteem (low implicit and high explicit self-esteem). More specifically, it might be possible that individuals with fragile self-esteem have more benefit from interventions that increase implicit self-esteem, because congruent high self-esteem (high implicit and explicit self-esteem) has been found to be an important predictor for psychological wellbeing (e.g., Jordan et al., 2003; Kernis et al., 2008). Furthermore, research into mechanisms that enhance the congruence between implicit and explicit self-esteem seems relevant. Recently, Koole et al. (2009) found that meditation appears to be effective to reduce implicit-explicit discrepancies (i.e., self-esteem). In addition, mindfulness training is aimed to enhance the clarity of thoughts, feelings, behaviors, and sensations of individuals (Brown et al., 2007). Since, Chiesa and Serretti (2009) showed that mindfulness training leads to decreased levels of stress, it might be of interest to examine the effect of mindfulness training on implicit-explicit discrepancies.

One limitation of the present study is that as a result of the cross-sectional design no conclusions with regard to causality can be drawn from this study. Furthermore, the sample consisted only of healthy young woman and future research should examine whether our findings can be generalized to other groups.

## Conflict of Interest Statement

The authors declare that the research was conducted in the absence of any commercial or financial relationships that could be construed as a potential conflict of interest.

## Footnotes

^1^The same sample as in the Creemers et al. (2012) study was used.

^2^As described in Creemers et al. (2012), variables of the conventional regression model and the discrepancy regression model need to be examined separately. These are separate models and, therefore, including the main effects of implicit and explicit self-esteem within the discrepancy regression model, would have led to a multicollinearity problem and to uninterpretable results.

## Appendix

Results of the conventional regression analyses and the discrepancy analyses when implicit self-esteem is measured with the Brief-IAT.

**Table A1:** **Hierarchical multiple regression analyses: associations of explicit self-esteem, implicit self-esteem (Brief-IAT), and the interaction between implicit and explicit self-esteem with suicidal ideation, depressive symptoms, and loneliness**.

	Suicidal ideation			Depressive symptoms			Loneliness		
	B	SE	β	B	SE	β	B	SE	β
**STEP 1**									
Implicit self-esteem	0.01	0.01	0.19†	0.06	0.08	0.06	−0.08	0.08	−0.08
Explicit self-esteem	−0.03	0.01	−0.45**	−0.72	0.08	−0.72**	−0.65	0.08	−0.65**
**STEP 2**									
Implicit self-esteem*	−0.01	0.01	−0.11	0.08	0.06	0.11	0.09	0.06	0.11
Explicit self-esteem									

*Suicidal ideation *R*^2^ = 0.18 in step 1 (*p* = 0.00); Δ*R*^2^ = 0.01 in step 2 (*p* = 0.26); depressive symptoms Δ*R*^2^ = 0.49 in step 1 (*p* = 0.00); Δ*R*^2^ = 0.01 in step 2 (*p* = 0.16); loneliness Δ*R*^2^ = 0.46 in step 1 (*p* = 0.00); Δ*R*^2^ = 0.01 in step 2 (*p* = 0.15); **p* < 0.05, ***p* < 0.01, †*p* = 0.053*.

**Table A2:** **Hierarchical multiple regression analyses: associations of the size of the discrepancy, direction of the discrepancy, and the interaction between the size of the discrepancy and the direction of the discrepancy with suicidal ideation, depressive symptoms, and loneliness**.

	Suicidal ideation			Depressive symptoms			Loneliness		
	B	SE	β	B	SE	β	B	SE	β
**STEP 1**									
Size of the discrepancy	0.03	0.01	0.35**	0.35	0.13	0.26**	0.09	0.14	0.06
Direction of the discrepancy	0.03	0.01	0.22*	0.65	0.19	0.33**	0.43	0.20	0.21*
**STEP 2**									
Size of the discrepancy*	0.04	0.02	0.53*	0.75	0.27	0.59**	0.79	0.29	0.61**
Direction of the discrepancy									

*Suicidal ideation *R*^2^ = 0.16 in step 1 (*p* = 0.00); Δ*R*^2^ = 0.05 in step 2 (*p* = 0.02); depressive symptoms *R*^2^ = 0.17 in step 1 (*p* = 0.00); Δ*R*^2^ = 0.06 in step 2 (*p* = 0.01); Loneliness *R*^2^ = 0.05 in step 1 (*p* = 0.09); Δ*R*^2^ = 0.07 in step 2 (*p* = 0.01); **p* < 05 ***p* < 01. Direction of the Discrepancy was dummy coded: 0 = fragile self-esteem; 1 = damaged self-esteem*.
